# Seo1p, a high-affinity, plasma membrane transporter of the γ-Glu-met dipeptide in yeasts and fungi

**DOI:** 10.1016/j.jbc.2025.108539

**Published:** 2025-04-25

**Authors:** Pratiksha Dubey, Md Shabbir Ahmad, Sunil Laxman, Anand K. Bachhawat

**Affiliations:** 1 Department of Biological Sciences, Indian Institute of Science Education and Research, SAS Nagar, Punjab, India; 2 DBT- Institute for Stem Cell Science and Regenerative Medicine (inStem), Bangalore, India

**Keywords:** peptide, γ-Glu dipeptides, glutathione, transporter, γ-Glu-met

## Abstract

γ-Glu dipeptides are ubiquitous in nature, and yet their metabolism and transport are poorly understood. Here we investigate this using the dipeptide γ-Glu-met in *Saccharomyces cerevisiae*. γ-Glu-met was efficiently utilized by *S. cerevisiae*, and using a transcriptomics approach, followed by a genetic screen, we identified Seo1p, an orphan transporter of yeast, as the transporter of γ-Glu-met. Uptake studies confirmed Seo1p as a high-affinity (Km = 48 μM), highly specific transporter of γ-Glu-met, as other analogs like n-Glu-met, γ-Glu-leu, γ-Glu-cys, γ-Glu-met-gly, methionine, and methionine sulfoxide were not transported by Seo1p. The expression of SEO1 was also repressed by these sulfur sources in the medium, but it was derepressed in the presence of γ-Glu-met. Seo1p homologs were present in yeast and fungi, and both *Candida auris* and *Candida albicans* were found to encode a functional Seo1p. The intracellular degradation of γ-Glu-met was investigated and found to be dependent on both the glutathione degrading cytosolic Dug2p/Dug3p complex, and the vacuolar γ-glutamyl transpeptidase, Ecm38p. Opt2p, a member of the oligopeptide transporter family, was also identified in the screen, and deletions in OPT2 led to an inability to grow on γ-Glu-met. However, Opt2p was not primarily involved in γ-Glu-met uptake. Its deletion affected vacuolar biogenesis, which interfered with the degradation of the peptide through Ecm38p. These studies demonstrate how organisms have evolved dedicated pathways for the uptake of these unusual peptides.

γ-Glutamyl peptides are a class of widely prevalent, low-molecular-weight peptides of 2 − 3 amino acids, where an N-terminal glutamic acid links to an amino acid or a peptide. Some of the γ-Glutamyl tripeptides are well known and extensively investigated. For example, the γ-Glutamyl tripeptide glutathione (γ-Glu-cys-gly), that is critical for redox and other functions, is very abundant in eukaryotes. The tripeptide homoglutathiones that include γ-Glu-cys-ser and γ-Glu-cys-β-ala have been found in considerable amounts in certain plants (1), and the glutathione analog, ophthalmic acid (γ-Glu-2-aminobutryl glycine), has been known for many years (2). Much less is known about the γ-Glutamyl di-peptides. Of these, the γ-Glutamyl di-peptide, γ-Glu-cys, which is a precursor of glutathione and is the thiol-equivalent of glutathione in many archaea, is the best studied (3). However, there are several γ-Glu-di-peptides that are ubiquitously present across the kingdoms of life, including mammals, plants, fungi, yeast, and bacteria. One study identified many different γ-Glu peptides that included γ-Glu-glu, γ-Glu-met, γ-Glu-phe, γ-Glu-tyr, γ-Glu-trp and γ-Glu-leu in cyanobacteria (4). Indeed, γ-Glu-di-peptides have been identified in many different organisms, including mammals (5, 6, 7, 8).

The γ-Glutamyl-di-peptides are thought to be synthesized through enzymatic side reactions of different enzymes that include γ-Glutamyl transpeptidase (GGT), γ-Glutamyl cysteine synthetase or γ-Glutamyl cysteinyl ligase (GCS or GCL), glutathione synthetase (GS), and glutaminase (1, 9, 10). In case of glutathione synthetase γ-Glutamyl cysteinyl ligase, limiting levels of cysteine (which is often observed in cells) result in alternate amino acids being accepted by the enzyme that leads to various γ-Glutamyl-dipeptide formations (11). Due to side reactions of various enzymes, the γ-Glutamyl-di-peptides have been considered metabolic byproducts of different enzymes, and their physiological significance was unclear for many years. However, recent evidence has suggested that γ-Glu-di-peptides have physiological roles in cells. Many γ-Glu-peptides impart kokumi taste to food by activating the extracellular calcium-sensing receptor (CaSR) (12). The γ-Glutamyl dipeptide γ-Glutamyl glutamate has an excitatory effect on synaptic transmission by activating N-methyl-D-aspartate receptors in human and rat (13). Other γ-GPs have anti-inflammatory, antioxidant, metal ion chelating, antitumor, and hypoglycemic effects (14), and some γ-Glutamyl-dipeptides may have therapeutic importance (15) and disease associations (9, 16, 17).

The γ-Glutamyl-dipeptides are very stable due to the γ-Glutamyl bond, making them resistant to peptidases, and their degradation requires specific enzymes. Mammalian cells and bacteria contain γ-Glutamyl-cyclotransferases (γ-GCT), breaking down a γ-Glu-amino acid into five-oxoproline and amino acid. However, these enzymes are absent in fungi and plants, so their metabolism in these organisms is not known. How these peptides traverse across the cell membrane and enter the cell is also not known in different organisms. In humans, multiple pathways have been postulated for the transport of these peptides across the intestinal epithelial cell monolayer. These include active transport mediated by the peptide transporter 1 (PepT1), the paracellular route through tight junctions, transcytosis, and passive transcellular diffusion. PepT1 can facilitate the transportation of a variety of small peptides, such as Gly-Sar, Trp-His, Gly-Pro, and Ile-Arg-Trp (18). However, while the transport of γ-Glu-Val as well as γ-Glu-S-(Me) C, γ-Glu-Leu *via* PepT1 has been suggested, γ-Glu-di-peptide transport by specific pathways has not yet been demonstrated.

Given this large knowledge gap, we have initiated a study on γ-Glu-di-peptides in yeast to investigate the transport and metabolism of a γ-Glu-di-peptide in *Saccharomyces cerevisiae*. To address this question, we chose γ-Glu-met as the peptide for two reasons. First, it has a physiological role in cells (19). Second, it enabled us to set up a simple genetic screen to facilitate the analysis. We exploited the *S. cerevisiae met15Δ* strain (an organic sulfur auxotroph), where we observed that γ-Glu-met could be used as a source of sulfur after its transport and cleavage to methionine. The known peptide transporter, Ptr2p, and the known glutathione/oligopeptide transporter, Hgt1p/Opt1p, did not have a role in its transport. Hence, we attempted to identify both the transporter of γ-Glu-met and the enzymes involved in its degradation. Our studies reveal that the degradation of γ-Glu-met occurs through the action of two glutathione-degrading enzymes, Dug2p/Dug3p in the cytoplasm and the Ecm38p γ-glutamyl transpeptidase in the vacuole. We further identify an orphan transporter of *S. cerevisiae*, Seo1p, as an evolutionarily conserved, specific high-affinity transporter of γ-Glu-met. This study substantially advances our understanding of how these ubiquitous and unusual dipeptides might be transported and metabolized.

## Results

### Yeast cells can efficiently utilize γ-Glu-met as a sulfur source through degradation by the Dug2p/Dug3p protein complex and Ecm38p

**Figure 1:**
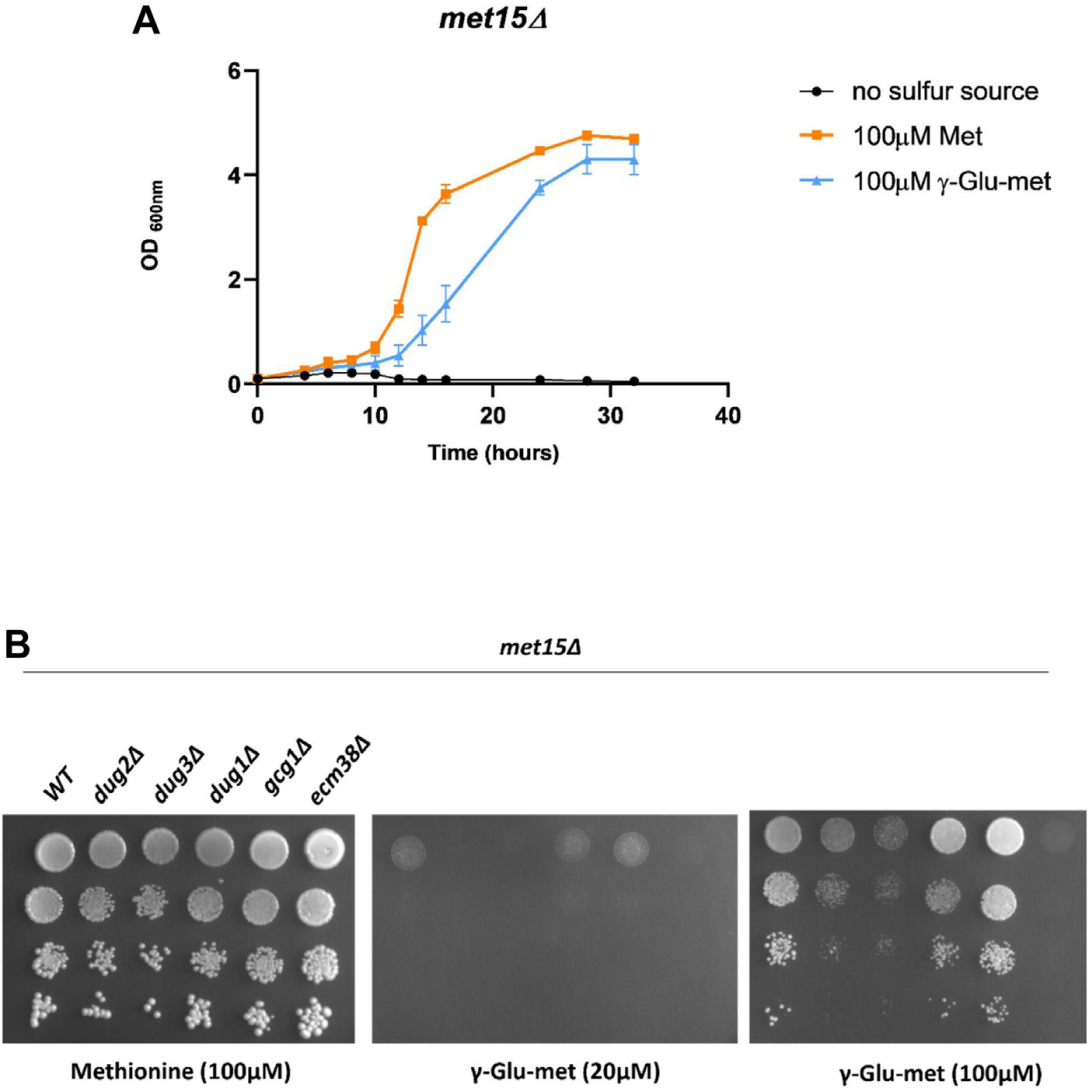
**Utilization of γ-Glu-met as a sulfur source in organic sulfur auxotrophic yeast cells (BY4741) and its breakdown by different enzymes.***A*, growth of *S. cerevisiae* BY4741 (*met15Δ*) on SD medium containing either γ-Glu-met, methionine, or no added organic sulfur in SD medium. The experiment was repeated three times and two representative data sets are plotted together, error bars represent the S.D. *B*, growth of *met15Δ* yeast cells bearing *ecm38Δ*, *dug1Δ, dug2Δ* or *dug3Δ* deletion on γ-Glu-met (20 μM and 100 μM) and 100 μM methionine (control). The yeast cells were grown overnight in minimal medium containing methionine as a sulfur source, washed and reinoculated in SD medium with no organic sulfur source till the exponential phase; Serial dilutions were spotted on the plates as described in methods. The photographs were taken after 72 h of incubation at 30 °C. The experiment was repeated three times, and a representative data set is shown.

To investigate the utilization of γ-Glu-met in yeast, we exploited the *met15Δ* strain, an organic sulfur auxotroph. The *met15Δ* strain exhibits growth only upon supplementation with an organic sulfur source such as methionine, cysteine, or glutathione. γ-Glu-met contains methionine, which can serve as a sulfur source. However, for this peptide to act as a sulfur source, it needs to be transported into the cell and subsequently degraded into glutamate and methionine. To test this possibility, we provided γ-Glu-met to *met15Δ* cells at a concentration of 100 μM. We observed that the *met15Δ* cells could efficiently utilize γ-Glu-met as a sulfur source (Fig. 1*A*). The generation time on methionine was 2.0 h, whereas on γ-Glu-met it was 4.0 h. This observation suggests the presence of a transport system for this peptide, as well as a degradation mechanism within the cells.

To find out the pathways involved, we initially evaluated previously known glutathione-degrading enzymes since glutathione is a γ-Glutamyl tripeptide, and previously known transporters for glutathione and peptides. There are four enzymes known to be involved in glutathione degradation; these include the Dug1 peptidase (20) that cleaves the cys-gly peptide and the Dug2p-Dug3p complex (21), Ecm38p (γ-Glutamyl transpeptidase) (22), and Gcg1p (Yeast Chac2) (23, 24), three enzymes capable of cleaving the γ-Glutamyl bond. The Dug2p-Dug3p complex is encoded by two different proteins, Dug2p and Dug3p, which combine to make a functional protein complex. The effect of deleting these different genes in a *met15Δ* background was evaluated. All these deletion strains were tested for γ-Glu-met utilization, based on assessing the eventual growth of cells. We find that *dug2Δ*, *dug3Δ*, and *ecm38Δ* were defective in utilizing the peptide, and *ecm38Δ* was the most severely affected in the utilization as seen on plates (Fig. 1*B*) and on liquid medium (Fig. S1). These data suggest that these enzymes participate in the degradation of γ-Glu-met to glutamate and methionine. Dug2p-Dug3p is a cytoplasmic complex previously known to be involved in GSH degradation, and the finding here suggests that the activity is not restricted to the tripeptide but it can also cleave γ-Glu-dipeptides. Ecm38p, on the other hand, is present on the vacuolar membrane, with its active site facing the vacuolar lumen.

### The putative membrane transporter, Seo1p, is involved in γ-Glu-met transport

**Figure 2:**
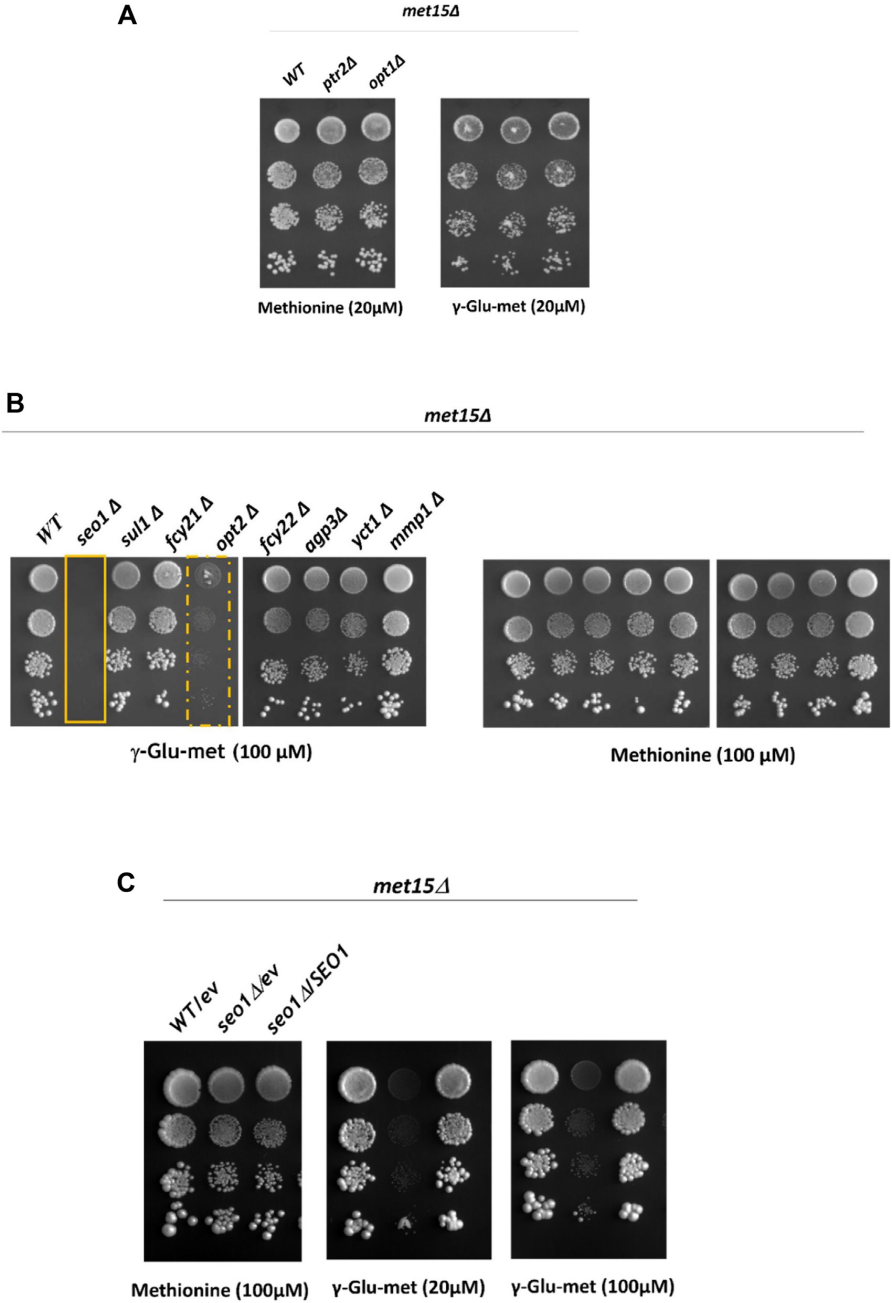
**Seo1p transporter is required for growth of deletion strains on γ-Glu-met.***A*, growth of deletions of the peptide transporter (*ptr2Δ*) and glutathione transporter (*opt1Δ*) in a *met15Δ* background on γ-Glu-met. *B*, growth of different transporter deletion strains in a *met15Δ* background on γ-Glu-met utilization; The yeast cells were grown overnight in minimal medium containing methionine as a sulfur source, washed and reinoculated in SD medium with no sulfur source. Cells were harvested at the exponential phase and serial dilutions were spotted on plate containing γ-Glu-met and methionine. The experiment was repeated three times, and a representative data set is shown (*C*). Growth of *seo1Δ* cells transformed with either vector or the SEO1gene on γ-Glu-met and methionine; the transformants were grown in minimal medium (-ura, +met) overnight, washed and reinoculated in SD (-ura, -met) medium. Cells were harvested at the exponential phase and serial dilutions were spotted on SD (-ura) plates containing γ-Glu-met and methionine. The photographs were taken after 72 h of incubation at 30 °C. The experiments were repeated three times, and a representative data set is shown.

In the yeast *S. cerevisiae*, peptide or oligopeptide uptake is driven by different transporters. PTR2 is currently the sole known di- and tripeptide transporter of *S. cerevisiae* (25), while HGT1/OPT1 is a glutathione/oligopeptide transporter (26, 27). When we evaluated the effect of loss of these two genes in the *met15Δ* background, we did not observe any growth defect even at 20 μM of γ-Glu-met (Fig. 2*A*). This suggests that these two transporters are not involved in the utilization of γ-Glu-met.

As none of the known transporters appeared to be involved in γ-Glu-met transport, we carried out transcriptomic analysis of *S. cerevisiae met15Δ* cells grown on γ-Glu-met as compared to methionine to identify if any putative transporters are upregulated in the presence of γ-Glu-met. Cells were grown to mid-log phase, harvested, and RNA isolated, and RNA seq analysis was done as described in the Materials and Methods section. The total read count for replicate 1 and replicate 2 of methionine-treated cells (control) was 15 million and 12 million, respectively, whereas in the case of γ-Glu-met-treated cells (test), the total read counts for replicate 1 and replicate 2 were 13 million and 16 million, respectively. Linear regression analysis done for the differentially expressed genes displayed a correlation coefficient value r = 1.00 and r = 0.99 for control and test, respectively, showing high reproducibility (Fig. S2). We observed that the sulfur-regulated genes were induced in γ-Glu-met-treated cells (Table S1), consistent with our expectation, since methionine is a repressing sulfur source relative to γ-Glu-met. We focused our attention, however, on the transporter genes (Table 1). Many of the upregulated transporters were also known sulfur-regulated proteins (28), and we asked if these might be involved in γ-Glu-met utilization by evaluating the growth on γ-Glu-met for deletions in a *met15Δ* background.

**Table 1:** Transporter genes upregulated in γ-Glu-met *versus* methionine as seen by RNA seq. *met15Δ* cells were grown on either γ-Glu-met or methionine as a sulfur source and harvested at 1 OD

Gene	log_2_Fold	*p* value	Function
SUL1	3.75	6.67E-35	High affinity sulfate permease of the SulP anion transporter family
SEO1	3.26	5.54E-29	Putative permease
YCT1	2.89	2.87E-23	High-affinity cysteine-specific transporter
AGP3	2.50	1.83E-18	Low-affinity amino acid permease
SUL2	2.25	5.45E-16	High affinity sulfate permease
MEP2	1.45	4.83E-07	Ammonium permease involved in regulation of pseudohyphal growth
VBA5	1.31	0.022912	Plasma membrane protein of the Major Facilitator Superfamily (MFS)
ATR2	1.22	0.00065	Putative boron transporter involved in boron efflux and resistance
FCY21	1.11	0.000123	Putative purine-cytosine permease
OPT2	1.04	0.000219	Oligopeptide transporter
MMP1	1.03	8.97E-05	High-affinity S-methylmethionine permease

RNA seq analysis was carried out to find out the upregulated transporters as described in the Experimental procedures. Only the genes which had a *p* < 0.05 and a log_2_ foldchange >1 were included in the list.

We observed that *seo1Δ* showed a complete growth defect on γ-Glu-met supplemented SD medium, while *opt2Δ* also showed a growth defect (Fig. 2*B*). As Seo1p is an uncharacterized transporter, we initially focused on SEO1. The *seo1Δ* phenotype could be rescued by complementation with a WT copy of SEO1 at 20 μM of γ-Glu-met (Fig. 2*C*).

**Figure 3:**
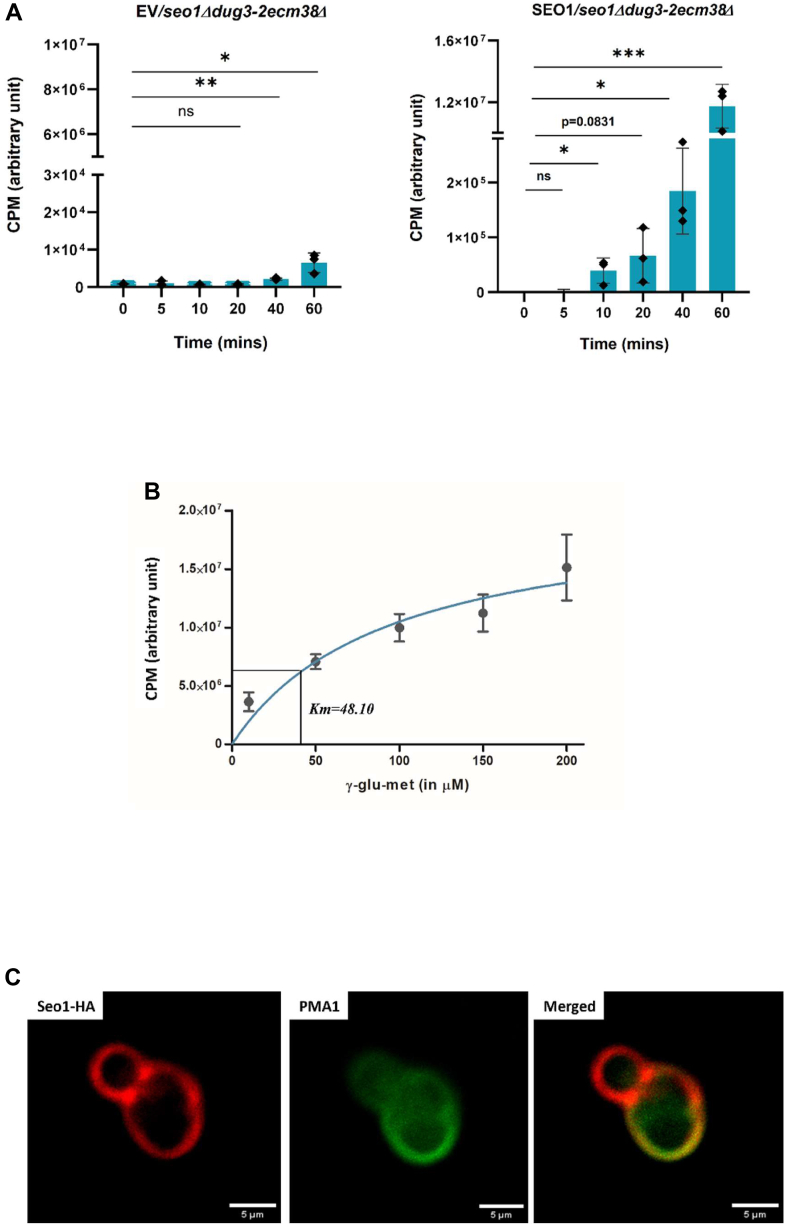
**Seo1p is a plasma membrane transporter of γ-Glu-met in yeast.***A*, γ-Glu-met uptake was measured using LC-MS/MS at different time intervals *i.e.* 5, 10, 20,40 and 60 min. *Left panel* shows the data with vector/*seo1Δmet15Δecm38Δdug3-2* strain. *Right panel* shows the data with SEO1 gene overexpression in *seo1Δmet15Δecm38Δdug3-2*. The graph shows a representative data set of three biological replicates. Error bars indicate mean ± S.D. (n = 3). ∗*p* < 0.05; ∗∗*p* < 0.01; ∗∗∗*p* < 0.001, ns nonsignificant (Student's *t* test). *B*, Km determination: Different concentrations of γ-Glu-met *i.e.* 10, 50, 100, 150 and 200 μM was used for the uptake (at 40 min time point) by SEO1 transformed in *seo1Δmet15Δecm38Δdug3-2*. The graph shows a data set of three biological replicates. Error bars indicate S.D. (n = 3). Km was determined (graph pad Prism), CPM is count per minute (*C*) cellular localization of SEO1: anti-HA antibody (*red*) detect SEO1-HA which is confined to plasma membrane and colocalizes (*merged*) with plamsa membrane marker anti-PMA1 antibody (*green*), scale bar: 5 μm.

To confirm if γ-Glu-met uptake required Seo1p, we carried out uptake studies, where intracellular γ-Glu-met levels were measured by targeted LC-MS/MS. For this study, the *seo1Δ met15Δ ecm38Δ dug3-2* strain, which is deficient in γ-Glu-met degradation (as described above), was used. *SEO1* (under a TEF promoter) was expressed in these strains. The SEO1 overexpression in the *seo1Δ met15Δ ecm38Δ dug3-2* strain (Fig. 3*A*) showed a time-dependent uptake of γ-Glu-met (100 μM), while negligible uptake was consistently observed with the control vector. A similar profile, although with lesser levels, was seen in the *seo1Δ* strain, which still had intact degradation pathways (Fig. S3). These data unambiguously reveal that SEO1 is involved in γ-Glu-met transport. Next, we determined the effective Km for γ-Glu-met transport by measuring uptake at fixed times in cells with different concentrations of γ-Glu-met, and found that an effective Km was 48 μM (Fig. 3*B*). This was very consistent with the rescue of growth seen with the growth of cells from 20 μM to 100 μM γ-Glu-met.

We further estimated the degradation of γ-Glu-met into glutamate and methionine (*i.e.* the ability of cells to metabolize γ-Glu-met). SEO1 was expressed from a TEF promoter in *dug3Δ met15Δ* and *ecm38Δ met15Δ* strains separately, and the accumulation of γ-Glu-met was monitored using LC-MS/MS. These two strains are defective in γ-Glu-met degradation (as concluded from our growth experiments). The only source of methionine in this strain is from the γ-Glu-met peptide, although glutamate may be derived from other pathways. We observed that these strains had significantly lower methionine levels, establishing the requirement of Dug3p/Dug2p complex and Ecm38p in γ-Glu-met degradation. Glutamate levels were also reduced in *ecm38Δ* and *dug3Δ* cells (in comparison to *met15Δ*); however, this was not as significant as the decrease in methionine levels (Fig. S4). This is expected, since substantial amounts of glutamate will be synthesized *de novo* by cells.

The physiological and uptake experiments suggested that Seo1p must be present at the plasma membrane. To confirm this and to assess its intracellular localization, we tagged SEO1 with a C-terminal HA-tag. The tagged clone was functional (Fig. S5), and the localization studies indicated that the protein was predominantly localized to the plasma membrane (as observed with colocalization with the PMA1, a plasma membrane ATPase) (Fig. 3*C*). Seo1p is a member of the Dal5p family of permeases. Members of this family protein have 12 transmembrane domains, and include the high-affinity cysteine transporter Yct1p, Dal5p, *etc.* SEO1 was first identified in a screen for mutants resistant to the methionine analog, ethionine sulfoxide, but the substrate for this transporter has remained elusive till now.

### Evaluation of different analogs and related compounds reveals Seo1p to be a specific transporter of γ-Glu-met

**Figure 4:**
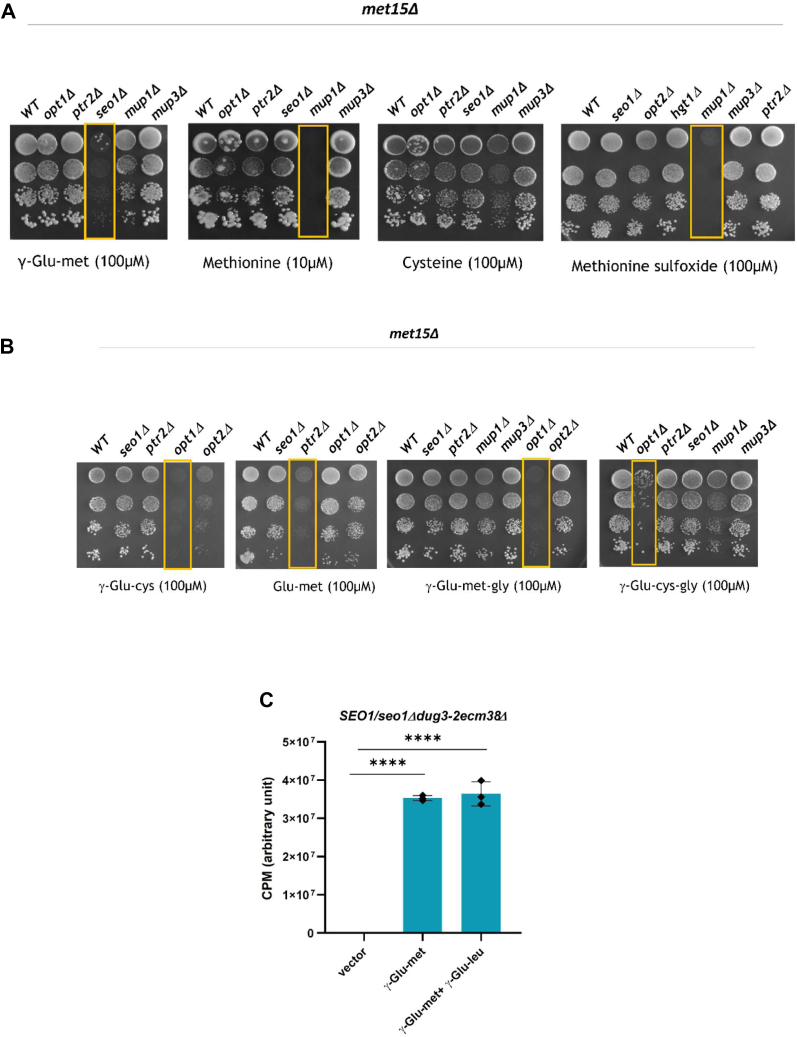
**Seo1p transports γ-Glu-met but no other analogous compounds.***A*, comparison of different deletion mutants for the utilization of either γ-Glu-met, methionine, cysteine, or methionine sulfoxide. *B*, comparison of different deletion mutants for the utilization of either γ-Glu-cys, Glu-met, γ-Glu-met-gly or glutathione. Yeast cells were grown overnight in minimal medium containing methionine as a sulfur source, washed and reinoculated in SD medium with no organic sulfur source. Cells were harvested at exponential phase and serial dilutions were spotted on plate containing different substrates at 100 μM. Only methionine plates were at 10 μM to evaluate *mup1Δ* since at higher concentrations secondary methionine transporters are functional. The photographs were taken after 72 h of incubation at 30 °C. The experiment was repeated three times, and a representative data set is shown (*C*) γ-Glu-leu at excess levels (400 μM) is unable to inhibit uptake of 50 μM γ-Glu-met by SEO1 in a *seo1Δmet15Δecm38Δdug3-2* background. The graph shows data for three biological replicates; error bars represent mean ± SD of values. ∗∗∗∗*p* < 0.0001 (Student's *t* test).

We next asked if other analogs of γ-Glu-met could also be transported by Seo1p and examined di-peptides that are structurally similar to γ-Glu-met. Since γ-Glu-cys is an intermediate of the glutathione cycle and is found to be present in yeast cells, we first examined how this peptide is utilized. We observed that it could be utilized as a sulfur source, but in this case, its transport was dependent on the glutathione transporter Opt1p/Hgt1p and not on Seo1p (Fig. 4*B*). Other γ-Glu peptides such as γ-Glu-leu and γ-Glu-his were also evaluated. To study the growth on γ-Glu-leu and γ-Glu-his, leucine and histidine auxotrophic strains were used for the respective peptides. However, the strains were unable to grow using these peptides. The reason for this could be either the absence of a transporter for γ-Glu-leu and γ-Glu-his or the absence of a degradation pathway to provide leucine and histidine for cell growth. Therefore, a competitive uptake inhibition of Seo1p was carried out using an excess of γ-Glu-leu (400 μM) along with γ-Glu-met (50 μM), and γ-Glu-met accumulation was estimated using LC-MS. However, cells did not show any reduction in γ-Glu-Met uptake in the presence of γ-Glu-Leu, suggesting that Seo1p is specific to γ-Glu-met uptake (Fig. 4*C*).

We also evaluated the tripeptide analog, γ-Glu-met-gly, using growth experiments. We observed that this peptide could support the growth of *seo1Δ* cells. The only deletion strain that was defective for growth of this tripeptide was the *hgt1Δ/opt1Δ* transporter that is otherwise essential for glutathione transport. These experiments indicated that the γ-Glu-met-gly tripeptide was not transported by the Seo1p transporter.

When we examined the peptide Glu-met (that contained the normal peptide bond rather than the γ-glutamyl bond), we observed that this peptide was able to support the growth of the *met15Δ* cells. However, the *seo1Δ* was not defective in growth on this peptide, and the only deletion strain that was defective for growth on this peptide was the strain deleted for the peptide transporter, *ptr2Δ*.

Since *seo1Δ* mutants were initially isolated as mutants resistant to ethionine sulfoxide, we also evaluated methionine sulfoxide as a possible substrate of Seo1p using growth experiments. As can be seen from the figure, the *seo1Δ* was not defective in the transport of methionine sulfoxide. In fact, only the high-affinity transporter for methionine, Mup1p, seemed to be primarily responsible for its utilization as a sulfur source, since only *mup1Δ* cells were defective in the growth of methionine sulfoxide as a substrate (Fig. 4*A*). Together, these studies along with the uptake studies, have led us to conclude that the Seo1p transporter is indeed very specific for the γ-Glu-met peptide.

### *Candida auris* and *Candida albicans* Seo1p can also transport γ-Glu-met

**Figure 5:**
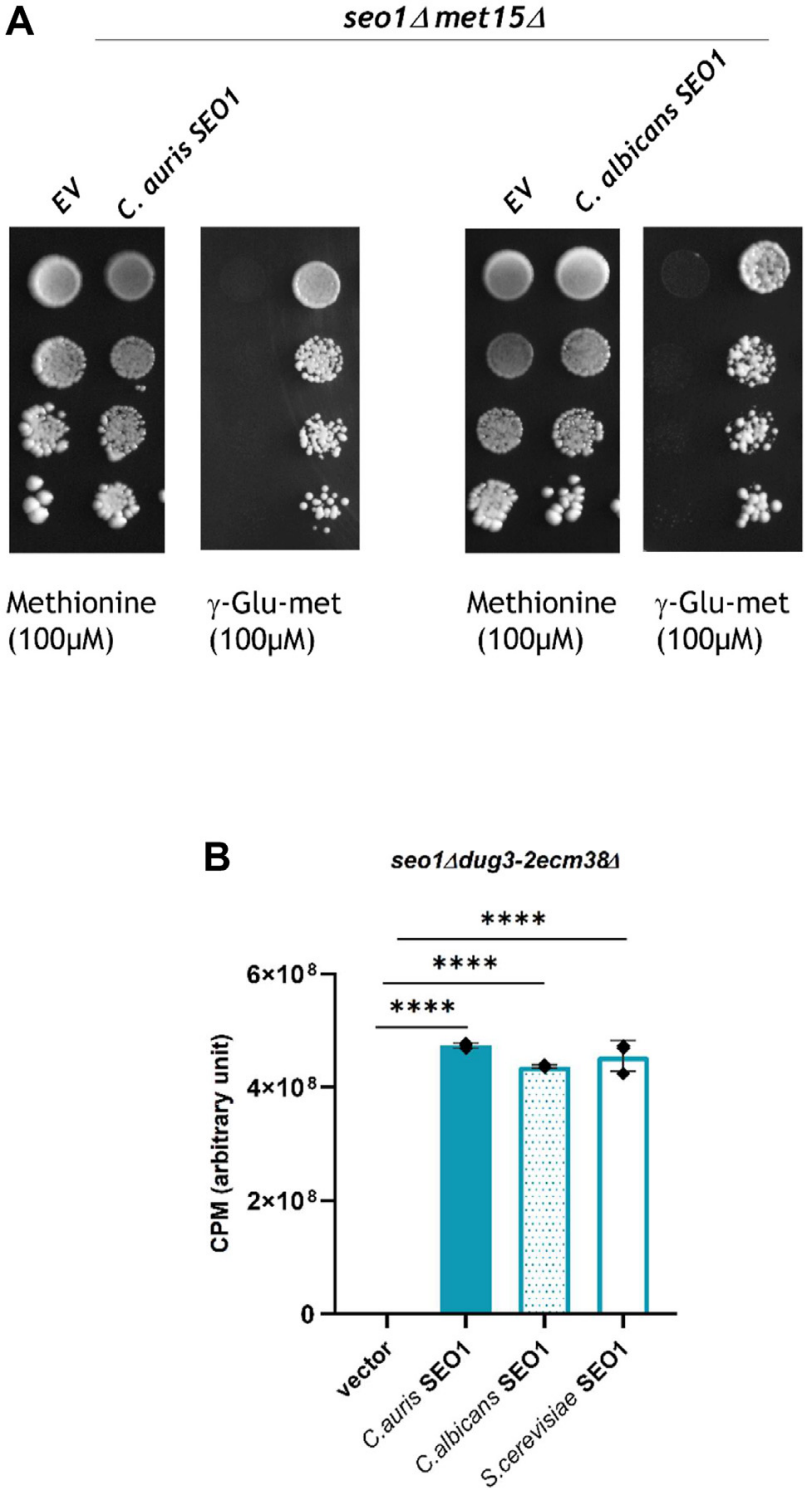
**Candida spp SEO1 orthologs encode functional γ-Glu-met transporters**. *A*, growth of the *S. cerevisiae seo1Δ* strain on γ-Glu-met is rescued upon expression of *Candida auris* and *Candida albicans* SEO1. The *S. cerevisiae seo1Δ* strain was transformed with either vector, *Candida auris* SEO1 or *Candida albicans* SEO1 and the transformants were grown in minimal medium (-ura, +met) overnight, cells were washed, inoculated in SD (-ura, -met) medium. Cells were harvested at the exponential phase and serial dilutions were spotted on SD (-ura) plates containing γ-Glu-met and methionine. The photographs were taken after 72 h of incubation at 30 °C. The experiment was repeated three times, and a representative data set is shown. *B*, confirmation of γ-Glu-met transport by *Candida auris* and *Candida albicans* SEO1 using LC-MS/MS. The graph was plotted for three biological replicates; error bars represent mean ± SD of values. ∗∗∗∗*p* < 0.0001 (Student's *t* test).

We examined the presence of orthologs of the *S. cerevisiae* Seo1p in other organisms. Seo1p orthologs appeared to be present in many yeasts and fungi, although we could not detect any orthologs in plants and humans. Putative orthologs that were identified in *Candida auris and Candida albicans* were cloned in the pRS416TEF plasmid and expressed in the *S. cerevisiae seo1Δ* strain (Fig. 5*A*). Uptake of γ-Glu-met (measured using LC-MS/MS) further confirmed the involvement of Seo1p homologs in γ-Glu-met transport (Fig. 5*B*).

### *opt2Δ* affects γ-Glu-met metabolism due to a defect in vacuolar biogenesis

In the initial screening of the different transporter deletions for growth on γ-Glu-met, in addition to *seo1Δ*, we had observed that deletions in the OPT2 transporter, *opt2Δ*, also showed a growth defect (Fig. 2*B*).

**Figure 6:**
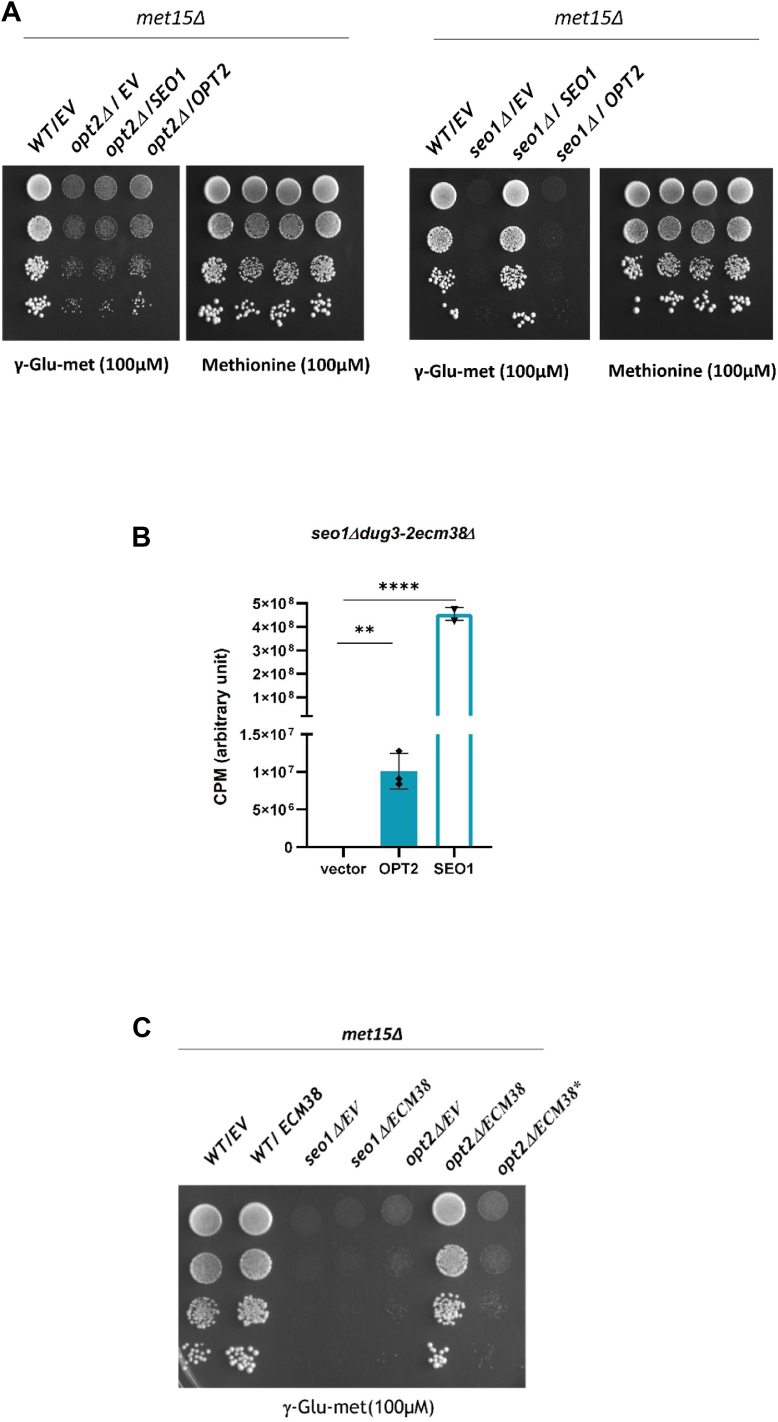
***opt2Δ* interferes with γ-Glu-met utilization not by any role in uptake but by its interference with Ecm38p activity through disruption of vacuolar morphology.***A*, *Left panel*: *opt2Δ* cells transformed with either vector, SEO1 or OPT2, and *Right panel*: *seo1Δ* cells transformed with either vector, SEO1 or OPT2 reveals that SEO1 and OPT2 do not cross rescue each other. The experiment was repeated three times, and a representative data set is shown. *B*, comparison of γ-Glu-met uptake by OPT2 relative to SEO1. The graph was plotted for three biological replicates; error bars represent mean ± SD of values. ∗∗*p* < 0.01; ∗∗∗∗*p* < 0.0001 (Student's *t* test). *C*, ECM38 expressed under TEF promoter rescues the defective phenotype of *opt2Δ* whereas an active site mutant of ECM38 (ECM38∗) was unable to rescue the defective phenotype of *opt2Δ* on γ-Glu-met. *seo1Δ* and *opt2Δ* strains were transformed with vector, ECM38 and ECM38∗. The transformants were grown in minimal medium (-ura, +met) overnight, cells were washed and grown in SD (-ura, -met) medium till the exponential phase. Cells were harvested and serial dilutions were spotted on SD (-ura) plates containing γ-Glu-met. The photograph was taken after 72 h of incubation at 30 °C. The experiment was repeated three times, and a representative data set is shown.

As we could clearly establish Seo1p as a plasma membrane transporter for γ-Glu-met, these observations of the *opt2Δ* defect on γ-Glu-met were surprising. Opt2p is a paralog of Opt1p/Hgt1p, a member of the oligopeptide transporter family. Opt1p/Hgt1p is the yeast glutathione transporter (26). However, the exact function of Opt2p has remained a mystery. An earlier study had localized an N-terminal GFP-tagged Opt2p to the punctate organelles that appeared to be peroxisomal and suggested functions as a peroxisomal and membrane glutathione transporter (29). However, other studies, including a more detailed investigation, revealed that OPT2 is primarily localized to Golgi and partly to the plasma membrane and have demonstrated that the *opt2Δ* has a severe vacuolar morphology (30). The primary function proposed was in regulating phospholipid asymmetry. To examine if Opt2p might in fact also be a second γ-Glu-met transporter, we examined if OPT2 overexpression could complement the *seo1Δ* phenotype. However, OPT2 failed to complement the *seo1Δ* to any significant extent (Fig. 6*A*). This was further confirmed by the uptake studies (Fig. 6*B*), where we observed only a very small uptake of γ-Glu-met by OPT2 overexpression. This did not explain the strong phenotype of *opt2Δ* on γ-Glu-met. As Opt2p was suggested to play a role in vacuolar biogenesis, we examined this phenotype afresh and did indeed observe disrupted vacuolar morphology with numerous small vacuoles in *opt2Δ* (Fig. S6). One possible explanation for the severe phenotype of *opt2Δ* on γ-Glu-met was therefore not due to any significant role in transport but because of defective vacuolar biogenesis. Ecm38p, γ-Glutamyl transpeptidase, which is involved in γ-Glu-met degradation, is a vacuolar enzyme. It is possible that the function of Ecm38p was disrupted in an *opt2Δ* background. To test this hypothesis, we decided to overexpress ECM38 with a strong promoter. We observed that the overexpression of ECM38 in the *opt2Δ* strain rescues the defective phenotype on γ-Glu-met. These observations demonstrate that OPT2 has only a minor role in γ-Glu-met transport and was primarily involved in the functioning of the degradation pathway. We also examined whether the overexpression of ECM38 was able to suppress the disrupted vacuolar morphology of *opt2Δ* cells. However, in *opt2Δ* cells bearing the pRSTEF416-ECM38 plasmid, we still observed that the cells contained a disrupted vacuolar morphology. This suggested that Ecm38p could only rescue the growth defect of *opt2Δ* on γ-Glu-met and not the other defects. To confirm if the suppression of the growth defect was indeed due to the enzymatic activity of Ecm38p, we used an active site mutant of Ecm38p (G494 → D494) (22) (Fig. 6*C*). However, expressing these active site mutants downstream of the TEF promoter did not lead to the growth of *opt2Δ* on γ-Glu-met. This confirmed that increasing the Ecm38p enzymatic activity was responsible for suppressing the *opt2Δ* phenotype on γ-Glu-met.

### SEO1 expression is sulfur-regulated

**Figure 7:**
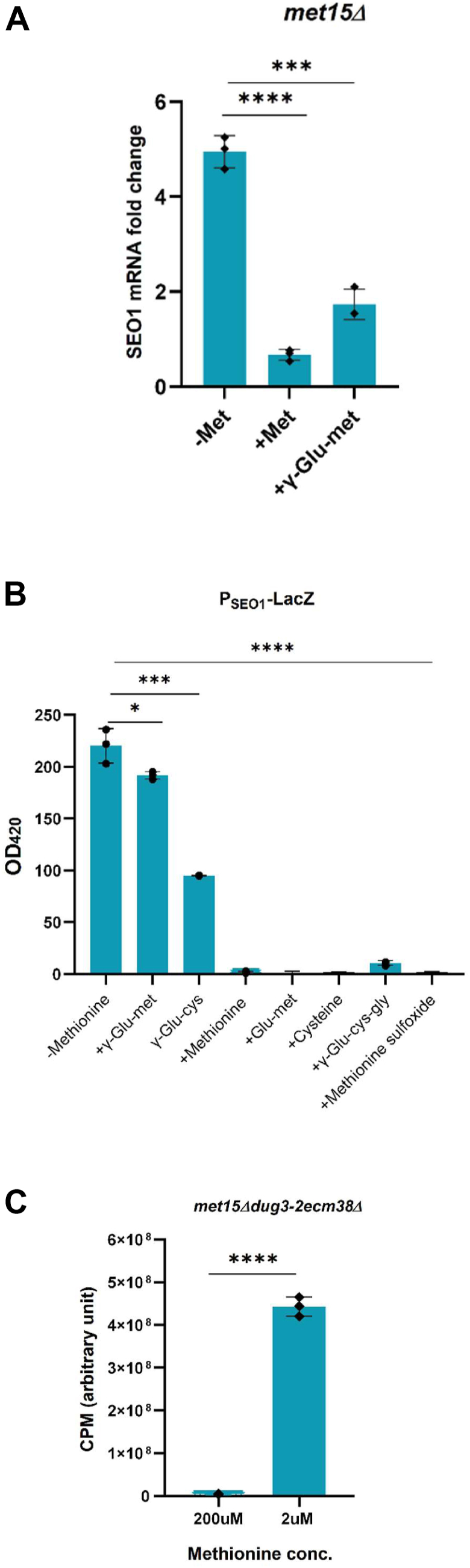
**SEO1 is a sulfur regulated protein.***A*, SEO1 mRNA expression analysis using q-RT-PCR. The experiment was repeated three times, and representative data were plotted for three technical replicates; error bars represent mean ± SD of values. ∗∗∗*p* < 0.001; ∗∗∗∗*p* < 0.0001. (Student's *t* test) *B*, β-galactosidase assay: SEO1 promoter sequence (673 bp)-lacZ fusion plasmid expressed in *met15Δ* strains and evaluated in the presence of different sulfur sources. The graph was plotted for three biological replicates; error bars represent mean ± SD of values. ∗*p* < 0.05 ∗∗∗*p* < 0.001; ∗∗∗∗*p* < 0.0001 (Student's *t* test). *C*, γ-Glu-met uptake in the presence of high and low concentrations of methionine monitored in *met15Δecm38Δdug3-2* strain using LC-MS/MS. The graph was plotted for three biological replicates; error bars represent mean ± SD of values. ∗∗∗∗*p* < 0.0001 (Student's *t* test).

In the transcriptomics data, we had observed that in growth on γ-Glu-met *versus* methionine, SEO1 was upregulated along with many other sulfur-regulated genes. SEO1 has also been observed in other genome-wide transcription profiles to be upregulated under sulfur limitation. To evaluate this, we first carried out qPCR studies. We observed that the SEO1 mRNA increased in low sulfur conditions (-met) whereas in the presence of methionine, the mRNA folds were reduced, which suggests that SEO1 expression is sulfur-regulated (Fig. 7*A*). We also cloned the SEO1 promoter (673 bp) upstream of the lacZ (β-galactosidase) gene in pLG699z vector to examine β-gal reporter activity in different sulfur conditions. SEO1 was induced under conditions where no methionine was added, whereas in the presence of methionine it was repressed. Similarly, SEO1 was also repressed when cysteine, GSH and even the normal dipeptide n-Glu-met was provided as a sulfur source. In contrast, SEO1 was induced in the presence of γ-Glu-met (Figs. 7*B* and S7). This regulation by sulfur was further confirmed at the uptake level by transport experiments, where we observed that cells grown in the presence of methionine (200 μM) led to a lack of ability to take up γ-Glu-met, while growth in low methionine (2 μM) in either *met15Δ* or *met15Δecm38Δdug3-2* background (with or without the degradation pathway) allowed the uptake of γ-Glu-met (Figs. 7*C* and S7). These data confirm that the expression of the SEO1geen is sulfur-regulated.

## Discussion

γ-Glu-di peptides occur ubiquitously; they can thus be used as a nutrient source by different organisms and can also serve as important metabolites to activate or inhibit pathways. In this study, we find that γ-Glu-met can be utilized by yeast as a sulfur source. The growth on γ-Glu-met was only a little slower than on methionine, suggesting that it is used as an efficient sulfur source. Investigating its degradation revealed that it is metabolized by two different enzymes; the glutathione-degrading enzyme Dug2p-Dug3p complex and the glutathione degrading enzyme Ecm38p. Interestingly, one of them is known to be cytosolic (Dug2p-Dug3p), acting on cytosolic pools, while the other is vacuolar (Ecm38p), acting on vacuolar pools.

What was most surprising in this study, however, was the observation that γ-Glu-met uptake occurs through a dedicated transporter, Seo1p, a previously uncharacterized transporter of the DAL5 family conserved amongst most yeasts and fungi. The high specificity and high affinity (Km for γ-Glu-met was in the micromolar range) was unexpected because there are no reports yet of γ-Glu-met being detected in yeasts. While establishing the specificity of Seo1p for γ-Glu-met by testing out other possible substrates, we also found out which transporters were involved in the transport of these other compounds. Thus, Mup1p, which is known as a high affinity methionine transporter, was also involved in the transport of methionine sulfoxide. The general di/tri peptide transporter Ptr2p was also found to be involved in transport of n-Glu-met. Further, the other tri peptide included in this study, γ-Glu-met-gly, was transported by the very well-known high affinity glutathione transporter Hgt1p.

SEO1 was first isolated in a screen for mutants resistant to ethionine sulfoxide toxicity where MUP1 and MUP3 were also isolated (31). The conclusion the authors could make at that time was that Seo1p does not transport methionine and that it is possibly sulfur regulated. Its isolation as an ethionine sulfoxide mutant further suggested a substrate similar in size or structure. All of these are consistent with γ-Glu-met as the substrate for this transporter. Thus, SEO1 which has for long remained a gene coding for an orphan transporter, has now finally a substrate and function assigned to it.

In this context, it is interesting to note that in a recent study, MFSD1, a lysosomal membrane protein was also recently deorphanized and shown to be a transporter of dipeptides (32). Although a variety of dipeptides were evaluated in this study, γ-glutamyl dipeptides were not examined. Since Glu-lys and His-glu were two of the most efficient substrates transported by MFSD1, given the findings reported here, it would be interesting to see if γ-glutamyl peptides could also be transported.

SEO1 expression was derepressed in the absence of sulfur sources cysteine, methionine, methionine sulfoxide and even n-Glu-met. Strikingly, γ-Glu-met was not a repressing source, while n-Glu-met was a strong repressor of SEO1. This is very consistent with the observation of Seo1p as a γ-Glu-met transporter. Importantly, the ability to transport γ-Glu-met by Seo1p is well conserved across budding yeast, and is not limited to *S. cerevisiae*, since the orthologs from distant pathogenic yeasts, *Candida auris* and *Candida albicans* were functional for γ-Glu-met transport. Thus, SEO1 seems to be an evolutionarily conserved transporter in the yeasts and fungi.

We also evaluated the role of the putative oligopeptide transporter Opt2p, a homolog of glutathione transporter (Hgt1p/Opt1p) in the utilization of γ-Glu-met. We initially looked at the transport capabilities of this protein. However, uptake data clearly indicated that Opt2p did not import γ-Glu-met significantly. Since the defect of *opt2Δ* for growth on γ-Glu-met was severe, we sought for an alternative explanation and probed the possibility that the reported role of OPT2 in vacuolar function could be important. According to previous reports, OPT2 has a role in phosopholipid asymmetry and affects the assembly of vacuole and maintenance of vacuolar morphology (30). We examined if disrupting vacuolar function might be in respect to the γ-Glu-met degrading enzyme Ecm38p which localizes to vacuolar membrane, with its active site facing the vacuolar lumen. While overexpressing this enzyme in an *opt2Δ* background could suppress the growth defect on γ-Glu-met, the disrupted vacuolar phenotype could not be suppressed by this overexpression and could explain the effects of opt2*Δ* as being due to a deficiency of the Ecm38p activity.

Why yeasts may have evolved a high-affinity γ-Glu-met transporter is puzzling. γ-Glu-met is present in human and mouse (19), but there is no report yet of its presence in yeast. A possible hypothesis for the presence of a γ-Glu-met transporter in yeast may lie in the fact that γ-Glu-met is a sulfur containing dipeptide. Sulfur-containing γ-Glutamyl peptides derivatives are widely found in plants (as a defense mechanism against virus, bacterial, microorganisms, insects, predators, and other plants (6, 33). It is possible that some fungi have evolved a transport mechanism for sulfur containing γ-Glu-di-peptide to salvage organic forms of sulfur thfrom the environment, since organic, reduced forms of sulfur are always limiting. Thus, a sulfur-regulated transporter for γ-Glu-met could be very beneficial in sulfur-limiting environments.

## Experimental procedures

### Chemicals and reagents

All chemicals used were of analytical reagent grade. Media components were purchased from Hi Media, Merck, and BD Difco. Oligonucleotides were purchased from Merck (Germany) and GCC (India). Restriction enzymes, Phusion polymerase, dNTPs, and other modifying enzymes were obtained from New England Biolabs. Gel-extraction kits and plasmid miniprep columns were obtained from QIAGEN or Thermo-Fischer Scientific. For initial experiments, γ-Glu-met was custom synthesized from Bachem, and for further experiments, γ-Glu-met and other γ-Glu-di-/tri-peptides were custom synthesized from GenScript (Biotech Desk Pvt. Ltd). Antibodies were purchased from AbCam and Cell Signaling Technologies.

### Strains, media and Growth

The *Escherichia coli* strain DH5a was used as a cloning host. The list of yeast strains used in this study is shown in Table S3. Yeasts were routinely maintained on YPD medium. The minimal medium contained YNB, glucose, and ammonium sulfate supplemented with the required amino acids and bases. Yeast DNA Isolation and Yeast Transformation—Yeast chromosomal DNA was isolated by the glass bead lysis method, and yeast transformations were carried out using the lithium acetate method (34).

#### Growth curve in liquid culture

The sulfur auxotrophic strain (*met15Δ*) was grown in complete synthetic medium overnight. Further, the inoculum (25 ml) was initiated at 0.01 OD_600_ on synthetic medium supplemented with 100 μM γ-Glu-met, 100 μM methionine (control) as sulfur source, and growth was monitored for 32 h. The data were plotted to obtain the generation time during logarithmic growth.

#### RNA sequencing

The AB6302 strain was grown in SD medium containing 100 μM methionine (control) and 100 μM γ-Glu-met (test) as a sulfur source. The mid-log phase cells were harvested, washed with water, and frozen. Total RNA was isolated using Qiagen RNeasy mini kit (Cat # 74106), RNA sequencing libraries were prepared with Illumina-compatible NEBNext Ultra II Directional RNA Library Prep Kit (New England BioLabs) were carried out by Genotypic Pvt.Ltd. Further, paired-end Illumina Next Generation Sequencing was performed at Genotypic Technology Pvt. Ltd. The data were deposited in NCBI (Accession no. PRJNA1212331).

#### Recombinant DNA

SEO1 (1782 bp) gene was cloned in pRS416TEF by PCR amplification followed by homologous recombination in yeast as described previously (35). The gene-specific primers carried overlapping sequences for pRS416TEF, and an internal restriction site Hind III and XhoI were also added in forward and reverse primers, respectively (Table S2). Two further sets of primers were also designed to amplify the vector in two different fragments carrying the overlapping sequences for homologous recombination. One fragment of the vector, called the CEN fragment, contains the CEN; another is called the URA fragment as it contains the URA marker. For homologous recombination, the CEN and URA fragments, along with the SEO1 gene fragment, carrying vector overlapping sequences, were transformed in yeast using the lithium acetate method and plated on SD-URA plates. A few colonies were picked up, and the desired recombinants were confirmed by PCR. The Plasmids were isolated after passage through *E. coli,* and digestion by the restriction enzymes HindIII and XhoI confirmed the presence of the clone. OPT2, OPT2-HA, and *Candida* SEO1 were also cloned using the same homologous recombination method, and the primers used are described in Table S2. *C. auris* and *C. albicans* have a slightly different genetic code in the LEU codon. The SEO1 gene of both these yeasts had a single codon that codes for leucine in *Candida albicans* and *Candida auris*, but serine in *S. cerevisiae* was cloned and expressed without correcting for the codon.

#### Growth assay by dilution spotting

For growth assay, the different strains were grown overnight in minimal ammonia medium without uracil and reinoculated in fresh medium to an OD_600_ of 0.1 without Uracil and methionine and grown for 6 h. The exponential-phase cells were harvested, washed with water, and resuspended in water to an OD_600_ of 0.2. These were serially diluted to 1:10, 1:100, and 1:1000. Of these cell resuspensions, 10 ml were spotted on minimal medium with different sulfur sources described as the sole organic sulfur source. The plates were incubated at 30 degrees for 3 days, and photographs were taken.

#### Cellular localization of SEO1 using microscopy

c-terminal HA tagged pRS416TEF-SEO1 was transformed in *seo1Δ* strain, these transformants were further grown in SD without uracil medium for localization experiments. Primary culture was grown overnight, and further secondary culture was started at 0.2OD and grown for 4 to 5 h. The cells in exponential phase were harvested and spheroplasts were made using zymolyase. The spheroplasts were washed with KPO4 buffer and fixed in 4% PFA. Further, permeabilization was done by using 0.1% Triton X and blocked with 1% BSA. Cells were further incubated with Anti-rabbit Anti-HA antibodies (1:1000 dilution) and Anti-mouse Anti-PMA1 (1:1000 dilution) antibodies at 4 degrees overnight. Next day, cells were washed with PBS and incubated with Anti-mouse Alexa flour 488 (1:500 dilution) and Anti-rabbit Alexa flour 647 (1:500 dilution) secondary antibodies for 2 h at RT. Finally, cells were washed with PBS and visualized under Nikon ti2 eclipse microscope.

#### Vacuolar morphology and microscopy

To visualize the vacuole, cells in log phase were stained with 8 μM FM4-64 (Cat #S6689) for 30 min, washed, and resuspended in the SD medium, and incubated for an additional 1 h at 30 °C (30). Cells were visualized under Nikon Ti2 Eclipse microscope.

#### Metabolite extraction and LC-MS/MS analysis

To measure the uptake of γ-Glu-met peptide by SEO1, *seo1Δ dug3-2 ecm38Δ* (AB6601) cells with SEO1 overexpression and empty vector (control) were grown in SD without uracil medium overnight and reinoculated in SD without uracil and methionine medium at 0.02 OD_600_ for 4 to 5 h. Cells in exponential phase were supplemented with 100 μm γ-Glu-met, metabolite quenching and cell harvesting were done at different time intervals *i.e.* 5, 10, 20, 40, and 60 min. The cells were pelleted down at 600*g* for 3 min, and metabolites were extracted, as described earlier (36). Briefly, 1 ml of ice-cold 10% methanol was added without disturbing the pellet (to quench metabolism) and further centrifuged at 600*g* for 3 min at 4 °C. Furthermore, 1 ml of 80% methanol (maintained at −45 °C) was added to the pellet, vortexed for 15 s, and incubated at −45 °C for 5 min for metabolite extraction. The tubes were vortexed (15 s) and centrifuged at 21,000*g* for 10 min at −5 °C. The supernatant (900 μl) was transferred into fresh tubes, recentrifuged at 21,000*g* for 10 min at −5 °C, and the supernatant was removed and dried using a SpeedVac. The samples were stored at −80 °C briefly before analysis by targeted LC-MS/MS to assess the γ-Glu-met uptake, expanding methods described earlier(36). We used Synergi TM 4-m Fusion-RP 80-Å (100, 4.6 mm) LC column for detection, and the indicated metabolites were estimated using an ABSciex QTRAP 6500 in triple-quadrupole mode. For kinetics experiments, 10, 50, 100, 150, and 200 μM γ-Glu-met was used for uptake, and cells were harvested at 40 min. A similar protocol was followed for uptake of γ-Glu-met by *candida* SEO1 and ScOPT2 in *seo1Δ dug3-2 ecm38Δ* strain at 40 min time point.

#### Q-RT-PCR to study SEO1 regulation

Yeast cells were grown with -met, +met and +γ-Glu-met supplementation and harvested in an exponential phase of growth. RNA was isolated from these cells using Acid-Phenol method and cDNA was prepared using Thermo Fischer Scientific RevertAid cDNA synthesis kit. For genes, the primer set listed in Table S2 were used with a final reaction volume of 5 μl containing using Maxima SYBR Green qPCR Master Mix (Fermentas). PCR conditions were 95 °C for 3 min, then 40 cycles consisting of denaturation at 95 °C for 10 s, annealing at 60 °C for 10 s and extension at 72 °C for 30 s, followed by the melting curve protocol with 10 s at 95 °C and then 60 s each at 0.5 °C increments between 65 °C and 95 °C. The reactions were performed in triplicate for each sample. The relative amounts of target gene expression for each sample were calculated using the Livak method formula 2-(ΔΔCT) against an endogenous control actin for genes. Finally, the fold change against the control gene is calculated and plotted. Data analysis was performed by Graph pad prism using Student's *t* test. The significance of differences between means were calculated at a 5% level (*p* < 0.05).

#### Construction of the SEO1 promoter–LacZ fusion constructs for the β-galactosidase reporter assay

The SEO1 promoter sequence (673 bp) was PCR amplified using oligonucleotides listed in Table S2. The PCR products were purified, digested with XhoI and BamHI, and cloned into pLG669z (37). pLG669z -SEO1-Promoter was transformed into the *met15Δ* (AB5000) strain, and the transformants were selected on minimal media plates without uracil. Further, these transformants were grown in the presence of different sulfur sources such as 200 μM of methionine, γ-Glu-met, GSH, methionine sulfoxide, cysteine, Glu-met and γ-Glu-cys, as indicated. The β-galactosidase reporter assay was carried out using standard protocols, and relative activity was presented as OD420.

#### Induction conditions and β-galactosidase assay

Fresh yeast transformants were used in all β-galactosidase experiments. The transformants were picked, grown overnight in minimal ammonia medium without uracil, and reinoculated in fresh minimal ammonia medium (without any organic sulfur source) to an initial OD_600_ of 0.2. They were grown for an additional 6 to 7 h to induce β-galactosidase. The cells were then harvested and washed twice in LacZ buffer (60 mm Na_2_HPO_4_7H_2_O, 40 mm NaH_2_PO_4_H_2_O, 10 mm KCl, 1 mm MgSO_4_7H_2_O, 0.27% 2-mercaptoethanol, pH 7) and taken for the reporter gene assay. β-Galactosidase activity was assayed in permeabilized yeast cells essentially as described previously (37). Briefly, the cells were resuspended in LacZ buffer, permeabilized by the addition of 50 ml chloroform and 20 ml SDS (0.1%). They were then vigorously vortexed for 20 s. These samples were equilibrated for 10 min at 30 and then o-nitrophenyl β-galactopyranoside was added sequentially to the reaction samples. β-Galactosidase units are given as OD_420_ × 1000 min1 ml1/OD_600_ at 30. The experiments were repeated with a minimum of three independent colonies.

### Statistical analysis

All uptake data have been analyzed using Student's *t* test with *p* value cut-off of 0.05 using GraphPad Prism. Standard kinetics-based Km determination was applied.

## Data availability

All data are contained within the manuscript. RNA sequencing data were deposited in NCBI (Accession no. PRJNA1212331).

## Supporting information

This article contains supporting information.

## Conflict of interest

The authors declare that they have no conflicts of interest with the contents of this article.
