# Supplementary material for: Seo1p, a high-affinity, plasma membrane transporter of the γ-Glu-met dipeptide in yeasts and fungi
Source: J Biol Chem. 2025 Apr 25;301(6):108539. doi: 10.1016/j.jbc.2025.108539 (PMC7617739; doi:10.1016/j.jbc.2025.108539)
Supplement: Supporting information [file mmc1.docx]

**Seo1p, a high affinity, plasma membrane transporter of the γ-Glu-met dipeptide in yeasts and fungi**

Pratiksha Dubey^1^, Md Shabbir Ahmad^2^, Sunil Laxman^2^, Anand K Bachhawat^1^

Supplementary Table S1

| **protein** | **log_2_Fold** | ***p* Value** | **function** |
| --- | --- | --- | --- |
| JLP1 | 4.18 | 6.49E-42 | Fe(II)-dependent sulfonate/alpha-ketoglutarate dioxygenase |
| PDC6 | 4.03 | 1.32E-40 | Minor isoform of pyruvate decarboxylase |
| SUL1 | 3.75 | 6.67E-35 | High affinity sulfate permease of the SulP anion transporter family |
| SEO1 | 3.26 | 5.54E-29 | Putative permease |
| BDS1 | 3.00 | 1.5E-24 | Bacterially-derived sulfatase |
| GRX8 | 2.97 | 2.24E-23 | Glutaredoxin that employs a dithiol mechanism of catalysis |
| YCT1 | 2.89 | 2.87E-23 | High-affinity cysteine-specific transporter |
| FMO1 | 2.87 | 3.01E-23 | Flavin-containing monooxygenase |
|  | 2.83 | 0.005049 | ORF , Uncharacterized |
| MET10 | 2.62 | 8.12E-20 | Subunit alpha of assimilatory sulfite reductase |
| MET3 | 2.52 | 1.68E-18 | ATP sulfurylase |
| AGP3 | 2.50 | 1.83E-18 | Low-affinity amino acid permease |
|  | 2.33 | 0.000614 | Protein of unknown function |
| MET16 | 2.25 | 1.22E-15 | 3'-phosphoadenylsulfate reductase |
| SUL2 | 2.25 | 5.45E-16 | High affinity sulfate permease |

Table S1: Top 15 upregulated genes (in the RNA Seq γ-Glu-met vs Methionine)

Supplementary Table S2: Primers used in the study

| **Name** | **Sequence (5’-3’)** |
| --- | --- |
| FP-SOE1-Frag | TTAGAAAGAAAGCATAGCAATCTAATCTAAGTTTTCTAGAaagcttATGTATTCAATTGT |
| RP-SOE1-Frag | GGAGGGCGTGAATGTAAGCGTGACATAACTAATTACATGActcgagTTATTTTTCATCAG |
| RP-SOE1-HA | ctcgagTTAAGCGTAATCTGGAACATCGTATGGGTATTTTTCATCAGATACTG |
| RP-HA-Frag | GGAGGGCGTGAATGTAAGCGTGACATAACTAATTACATGActcgagTTAAGCGTAATCTG |
| FP-OPT2-Frag | TTAGAAAGAAAGCATAGCAATCTAATCTAAGTTTTCTAGAaagcttATGAGTGAAACAGT |
| RP-OPT2-HA | TTAAGCGTAATCTGGAACATCGTATGGGTATGGATAGTGTCCTGGAG |
| FP-*C.alb*-SEO1-Frag | TTAGAAAGAAAGCATAGCAATCTAATCTAAGTTTTCTAGAaagcttatgtctttcttttc |
| RP-*C.alb*-SEO1-Frag | GGAGGGCGTGAATGTAAGCGTGACATAACTAATTACATGActcgagctatttttctgatt |
| FP-*C.aur*-SEO1-Frag | TTAGAAAGAAAGCATAGCAATCTAATCTAAGTTTTCTAGAaagcttatgcttgagtcttt |
| RP-*C.aur*-SEO1-Frag | GGAGGGCGTGAATGTAAGCGTGACATAACTAATTACATGActcgagtcactgtttggtaa |
| FP-SEO1- Pro- XhoI | ATCCTActcgagGATACAAACTTTATCTATAATATCG |
| RP-SEO1- Pro- BamHI | ATCCTAggatccCATGTATGTTATTTATATATGCAGTAG |
| FP-SEO1-RT | TTGTGTCTGTCGTAGTGGGC |
| RP-SEO1-RT | GTTGTATGGGTCACCTGGCA |
| FP_416CEN | TCTAGAAAACTTAGATTAGATTGC |
| RP_416CEN | CTTCTGTTCGGAGATTACCGAATC |
| FP_416Ura | TCGCGCGTTTCGGTGATGAC |
| RP_416Ura | CTCGAGTCATGTAATTAGTTATGT |

Supplementary Table S3: Strains used in the study

| Strain | Genotype |
| --- | --- |
| AB460 | E.coli DH5α |
| AB5000 | BY4741;MATa;his3Δ1;leu2 Δ0;met15Δ0;ura3Δ0 |
| AB2067 | BY4741;MATa;his3Δ1;leu2Δ 0;met15Δ 0;ura3Δ 0;Seo1Δ::KanMX |
| AB2138 | BY4741;MATa;his3Δ1;leu2Δ 0;met15Δ 0;ura3Δ 0;Opt2Δ::KanMX |
| AB1417 | BY4741;MATa;his3Δ1;leu2Δ 0;met15Δ 0;ura3Δ 0;Ptr2Δ::KanMX |
| AB817 | BY4741;MATa; his3Δ1;leu2Δ 0;met15Δ 0;ura3Δ 0; Hgt1Δ::LEU2 |
| AB6302 | BY4741;MATa; his3Δ1;leu2Δ 0;met15Δ0;ura3Δ0; Oxp1Δ::KanMX |
| AB2743 | BY4741;MATa;his3Δ1;leu2Δ0;met15Δ0;ura3Δ0;Gcg1Δ::KanMX |
| AB1654 | BY4741;MATa;his3Δ1;leu2Δ0;met15Δ0;ura3Δ0;Dug1Δ::KanMX |
| AB1596 | BY4741;MATa;his3Δ1;leu2Δ0;met15Δ0;ura3Δ0;Dug2Δ::KanMX |
| AB1615 | BY4741;MATa;his3Δ1;leu2Δ0;met15Δ0;ura3Δ0;Dug3Δ::KanMX |
| AB1083 | BY4741;MATa;his3Δ1;leu2Δ0;met15Δ0;ura3Δ0;Ecm38Δ::KanMX |
| AB1839 | BY4741;MATa;his3Δ1;leu2Δ0;met15Δ0;ura3Δ0;Mup1Δ::KanMX |
| AB1844 | BY4741;MATa;his3Δ1;leu2Δ0;met15Δ0;ura3Δ0;Mup3Δ::KanMX |
| AB2356 | BY4741;MATa;his3Δ1;leu2Δ0;met15Δ0;ura3Δ0;Sul1Δ::KanMX |
| AB2060 | BY4741;MATa;his3Δ1;leu2Δ0;met15Δ0;ura3Δ0;Agp3Δ::KanMX |
| AB3084 | BY4741;MATa;his3Δ1;leu2Δ0;met15Δ0;ura3Δ0;Yct1Δ::His3 |
| AB1530 | BY4741;MATa;his3Δ1;leu2Δ0;met15Δ0;ura3Δ0;Mmp1Δ |
| AB6107 | BY4741;MATa;his3Δ1;leu2Δ0;met15Δ0;ura3Δ0;Fcy21Δ |
| AB6108 | BY4741;MATa;his3Δ1;leu2Δ0;met15Δ0;ura3Δ0;Fcy22Δ |
| AB6601 | MATα;his3Δ1;leu2Δ0;met15Δ0;ura3Δ0;LysΔ;ecm38Δ::KANMX2 dug3-2; Seo1Δ::His3 |

**
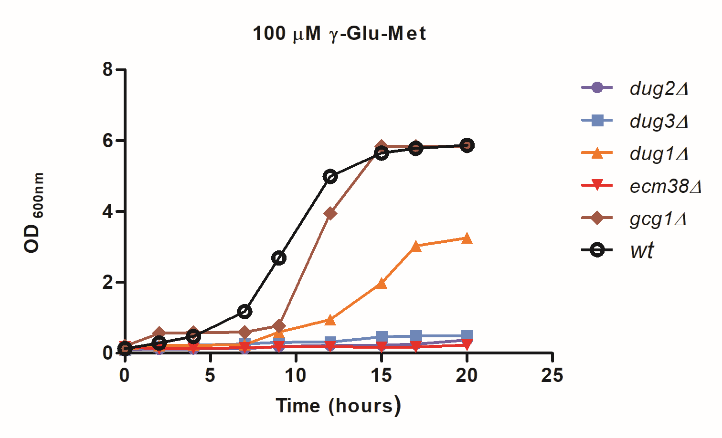
**

Figure S1: Growth of mutants defective in different degradation pathways (*dug1Δ; dug2Δ; dug3Δ*;*ecm38Δ* and *gcg1Δ*) on γ-Glu-met and the comparison with WT. All strains are in *met15Δ* background.


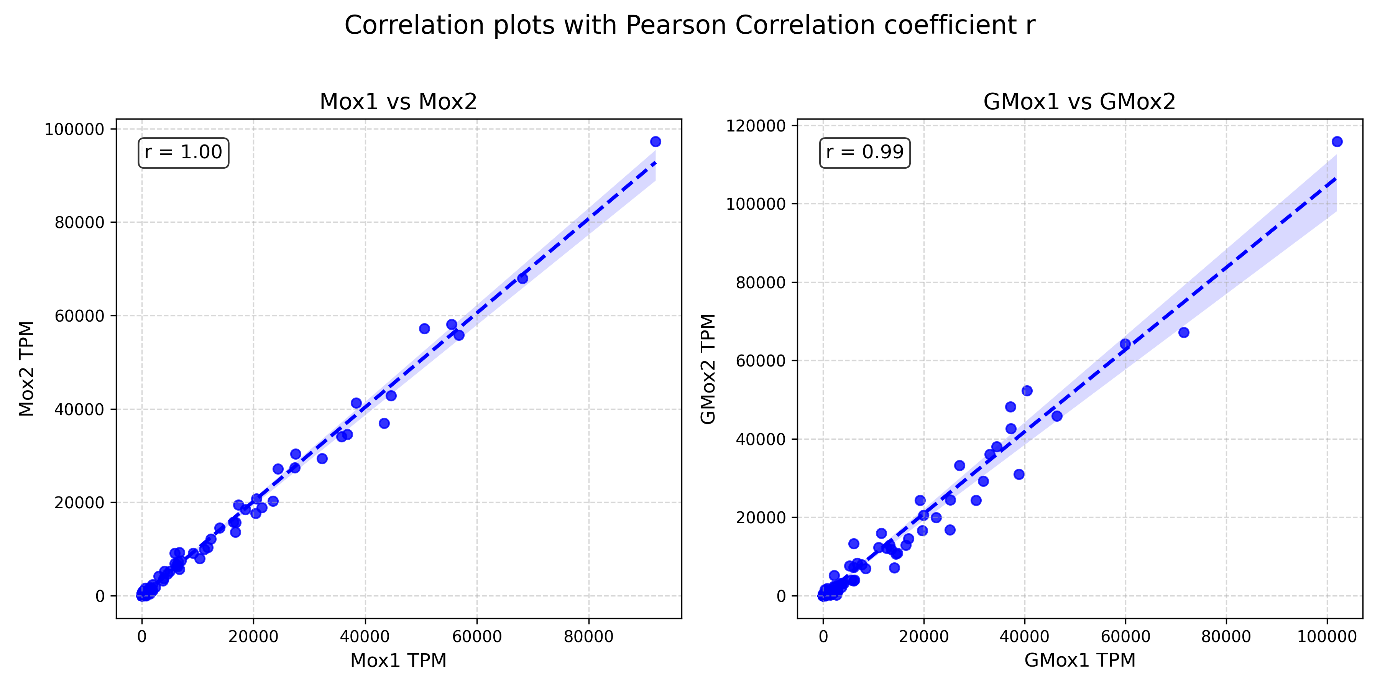


Figure S2: Correlation analysis of both replicates of transcriptomics data; cells grown in methionine (left panel) and γ-Glu-met (right panel).


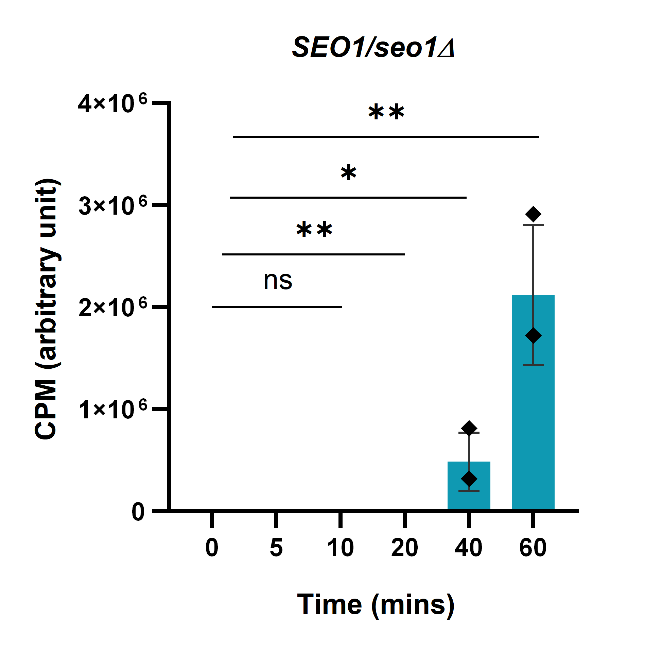


Figure S3: γ-Glu-met uptake by SEO1 in *seo1Δ* background: uptake was measured using LC-MS/MS at different time intervals i.e. 5, 10, 20,40 and 60 min in *seo1Δ* strain transformed with SEO1. The graph shows a representative data set of two biological replicates. Error bars indicate mean ± S.D. *, *p*< 0.05; **, *p* < 0.01; ns-non significant (Student’s t-test).


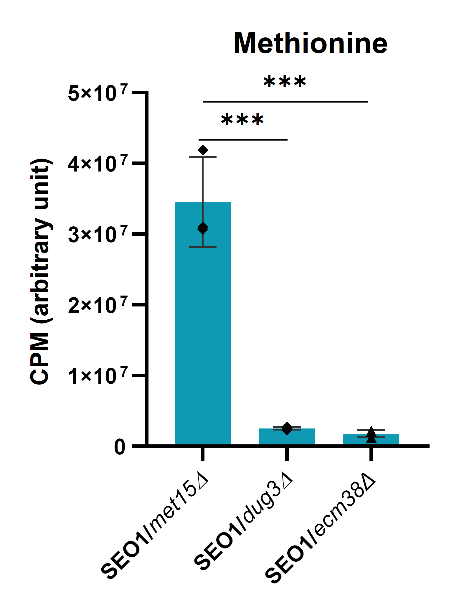

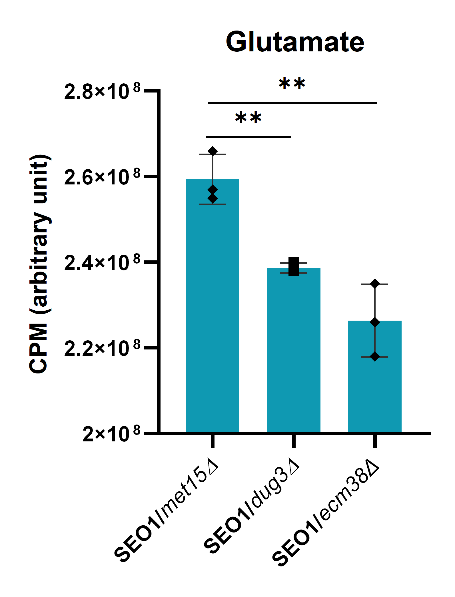


Figure S4: γ-Glu-met degradation by Ecm38p and Dug3p assessed by methionine and glutamate levels. *met15Δ,* *met15Δecm38Δ* and *met15Δdug3Δ* were transformed with SEO1 and the glutamate and methionine levels were assessed by LC-MS/MS at 40mins. The graph shows a representative data set of three biological replicates. Error bars indicate mean with S.D. (n =3). **, *p* < 0.01; ***, *p* < 0.001. (Student’s t-test)


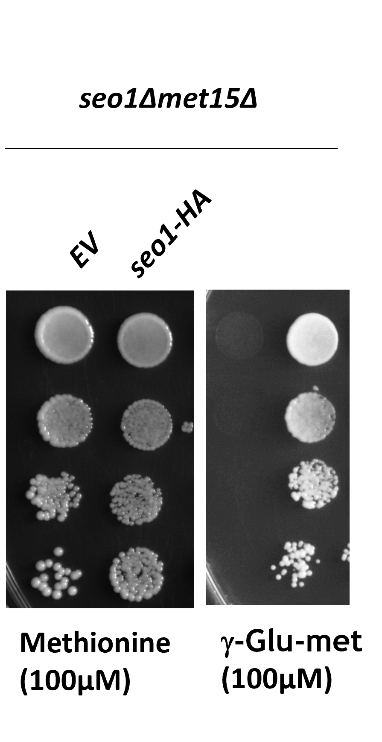


Figure S5: Assesment of functionality of SEO1-HA clone: C-terminally HA-tagged SEO1-HA clone or the vector was transformed into *seo1Δ* strain. The transformants were grown in minimal medium (-ura, +met) overnight, cells were washed and grown in SD (-ura, -met) medium till the exponential phase. Cells were harvested and serial dilutions were spotted on SD (-ura) plates containing γ-Glu-met and methionine. The photographs were taken after 72h of incubation at 30°C.


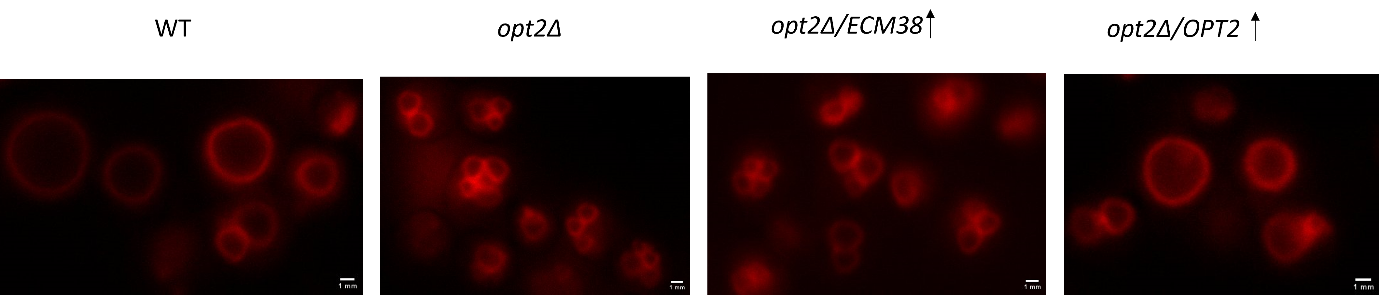


Figure S6: *opt2Δ* leads to vacuolar fragmentation:Vacuolar morphology of *opt2Δ* as seen using FM4-64 dye which intercalates in plasma membrane and through vesicular transport gets incorporated in vacuolar membranes. WT cells showed intact vacuolar morphology whereas in *opt2Δ* cells vacuoles are fragmented, Overexpression of OPT2 show a partial rescue of this phenotype. Overexpression of ECM38 did not show any rescue of the fragmented morphology. (Scale bar:1mm)


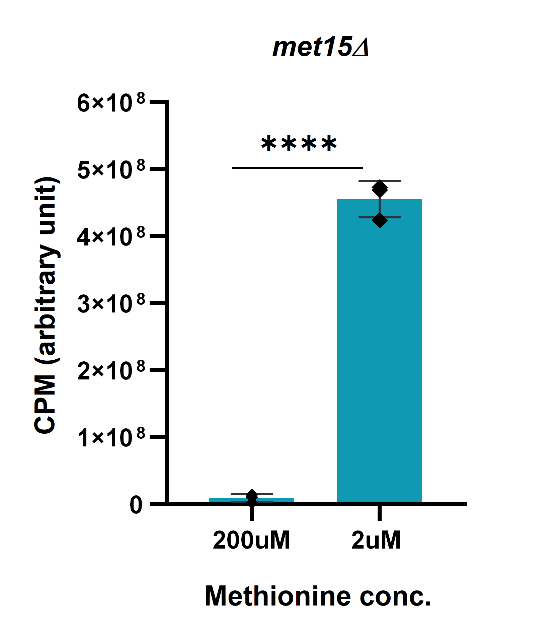


Figure S7: γ-Glu-met uptake is repressed when cells are grown on methionine in a genetic background with intact degradation pathway: γ-Glu-met uptake in the presence of high and low concentrations of methionine monitored in *met15Δ* strain using LC-MS/MS. The graph was plotted for three biological replicates; error bars represent mean± SD of values. ****, *p* < 0.0001. (Student’s t-test).
